# Mutations in the *Polycomb* Group Gene *polyhomeotic* Lead to Epithelial Instability in both the Ovary and Wing Imaginal Disc in *Drosophila*

**DOI:** 10.1371/journal.pone.0013946

**Published:** 2010-11-11

**Authors:** Pierre Gandille, Karine Narbonne-Reveau, Elisabeth Boissonneau, Neel Randsholt, Denise Busson, Anne-Marie Pret

**Affiliations:** 1 Centre de Génétique Moléculaire (FRE 3144), Centre National de la Recherche Scientifique, Gif-sur-Yvette, France; 2 Institut de Biologie du Développement de Marseille-Luminy (UMR 6216), Centre National de la Recherche Scientifique/Université de la Méditérannée Aix-Marseille II, Marseille, France; 3 Institut Jacques Monod (UMR7592), Centre National de la Recherche Scientifique/Université Pierre et Marie Curie-Paris VI, Université Denis Diderot-Paris VII, Paris, France; 4 Systematique Adaptation Evolution (UMR7138), Université Pierre et Marie Curie Paris VI, Paris, France; 5 Laboratoire de Biologie du Développement (UMR7622), Centre National de la Recherche Scientifique/Université Pierre et Marie Curie-Paris VI, Paris, France; 6 Université de Versailles-St Quentin, Versailles, France; St George's University of London, United Kingdom

## Abstract

**Background:**

Most human cancers originate from epithelial tissues and cell polarity and adhesion defects can lead to metastasis. The Polycomb-Group of chromatin factors were first characterized in *Drosophila* as repressors of homeotic genes during development, while studies in mammals indicate a conserved role in body plan organization, as well as an implication in other processes such as stem cell maintenance, cell proliferation, and tumorigenesis. We have analyzed the function of the *Drosophila* Polycomb-Group gene *polyhomeotic* in epithelial cells of two different organs, the ovary and the wing imaginal disc.

**Results:**

Clonal analysis of loss and gain of function of *polyhomeotic* resulted in segregation between mutant and wild-type cells in both the follicular and wing imaginal disc epithelia, without excessive cell proliferation. Both basal and apical expulsion of mutant cells was observed, the former characterized by specific reorganization of cell adhesion and polarity proteins, the latter by complete cytoplasmic diffusion of these proteins. Among several candidate target genes tested, only the homeotic gene *Abdominal-B* was a target of PH in both ovarian and wing disc cells. Although overexpression of *Abdominal-B* was sufficient to cause cell segregation in the wing disc, epistatic analysis indicated that the presence of Abdominal-B is not necessary for expulsion of *polyhomeotic* mutant epithelial cells suggesting that additional POLYHOMEOTIC targets are implicated in this phenomenon.

**Conclusion:**

Our results indicate that *polyhomeotic* mutations have a direct effect on epithelial integrity that can be uncoupled from overproliferation. We show that cells in an epithelium expressing different levels of POLYHOMEOTIC sort out indicating differential adhesive properties between the cell populations. Interestingly, we found distinct modalities between apical and basal expulsion of *ph* mutant cells and further studies of this phenomenon should allow parallels to be made with the modified adhesive and polarity properties of different types of epithelial tumors.

## Introduction

The development of multicellular organisms and homeostasis in the adult require the organization of cells into layers or epithelia. Epithelium formation and integrity are ensured via cell-cell adhesion mediated by formation of several specialized junctions that subdivide and polarize each epithelial cell into an apical and a basolateral membrane domain [1], [2], [3]. The molecular mechanisms underlying apico-basal cell polarization and cell-cell adhesion are evolutionary conserved among animals. The best characterized junctions are the apical adherens junctions composed of E-cadherin, localized at the cell membrane and able to form direct homophilic bonds, and β-catenin, which links the intercellular domain of E-cadherin to α-catenin, the latter interacting directly with the actin cytoskeleton [4], [5], [6], [7]. In the basal domain of epithelial cells, members of the integrin family are present and allow adhesion between different layers of cells via their binding to the extracellular matrix [8]. Dynamic intercellular adhesion is fundamental both for the recognition and assembly of cells with similar properties and for the segregation of cells into distinct populations [9], [10], [11]. However the link between developmental signals regulating adhesion molecule dynamics for proper epithelial organization remains poorly understood. Importantly, most human cancers originate from epithelial tissues and cell adhesion and polarity defects participate significantly to tumor progression and metastasis.

*polyhomeotic* (*ph*) is a member of the *Polycomb* group (*PcG*) genes [12], [13]. PcG proteins were first characterized in *Drosophila* where they have been shown to be required for the maintenance of a repressed state of target gene transcription, via multimeric protein complexes affecting chromatin structure [14], [15]. Although their best-documented role is the determination of segment identity along the anterior-posterior axis during embryogenesis via epigenetic regulation of homeotic genes, it is becoming clear that PcG proteins in mammals and in *Drosophila* are involved in many other processes, including cell proliferation [16], [17], [18], [19], maintenance of stem cell and differentiated cell identities [20] and cancer [21].

Previous analysis of *ph* gene function carried out in the *Drosophila* wing imaginal disc indicated that *ph* loss of function clones are expulsed from the epithelial layer, surviving into adulthood where they form vesicles maintaining epithelial characteristics [22], [23], [24]. In these studies, many different developmental genes were shown to be deregulated in *ph* mutant wing discs, including *engrailed*, *hedgehog*, and *decapentaplegic*, as well as several homeotic genes such as *Abdominal-B* and *Ultrabithorax*, but no functional connection has been demonstrated between these targets and the *ph* expulsion phenotype. Here, we present results indicating that the expulsion phenotype associated with *ph* mutations can be extended to a second model epithelium in *Drosophila*, the follicular epithelium of the ovary. Indeed, in the course of a screen to identify genes with somatic function in ovarian follicle formation during early oogenesis we identified *polyhomeotic* [25]. In the present study, we show that induction of *ph* loss of function follicular cell clones leads to progressive expulsion of mutant cells from the follicular epithelium as in the wing imaginal disc. We have characterized more precisely the expulsion phenotype of the *ph* mutant clones in both the wing disc and the ovarian follicular epithelia. The expulsion of *ph* mutant cells in both model epithelia is associated with cell polarity defects and, in particular, with specific modifications of apical adherens junctions. However, different modalities of expulsion, between tissues and even within a given tissue, were observed including apical vs. basal expulsions and reorganization vs. complete diffusion of apical/basal markers. Interestingly, *ph* overexpression clones also segregated from the rest of the epithelium indicating that it is likely the juxtaposition of cells with different levels of PH that leads to epithelial instability. In order to identify PH targets common to both the wing and ovary models, we tested several known targets and found that *Abdominal-B* (*Abd-B*), a homeotic target of PH during embryogenesis and wing development [22], [26] is also a target of PH in the ovarian follicular epithelium. Interestingly, ectopic expression of *Abd-B* in the wing disc also caused segregation of mutant and wild-type cells. However, epistatic analysis showed that the *ph* expulsion phenotype is not rescued upon downregulation of *Abd-B* indicating that other, as yet unidentified, PH targets are implicated in the epithelial instability provoked by *ph* mutations.

## Results

### *ph* mutant cells are expulsed basally from the ovarian follicular epithelium

**Figure 1:**
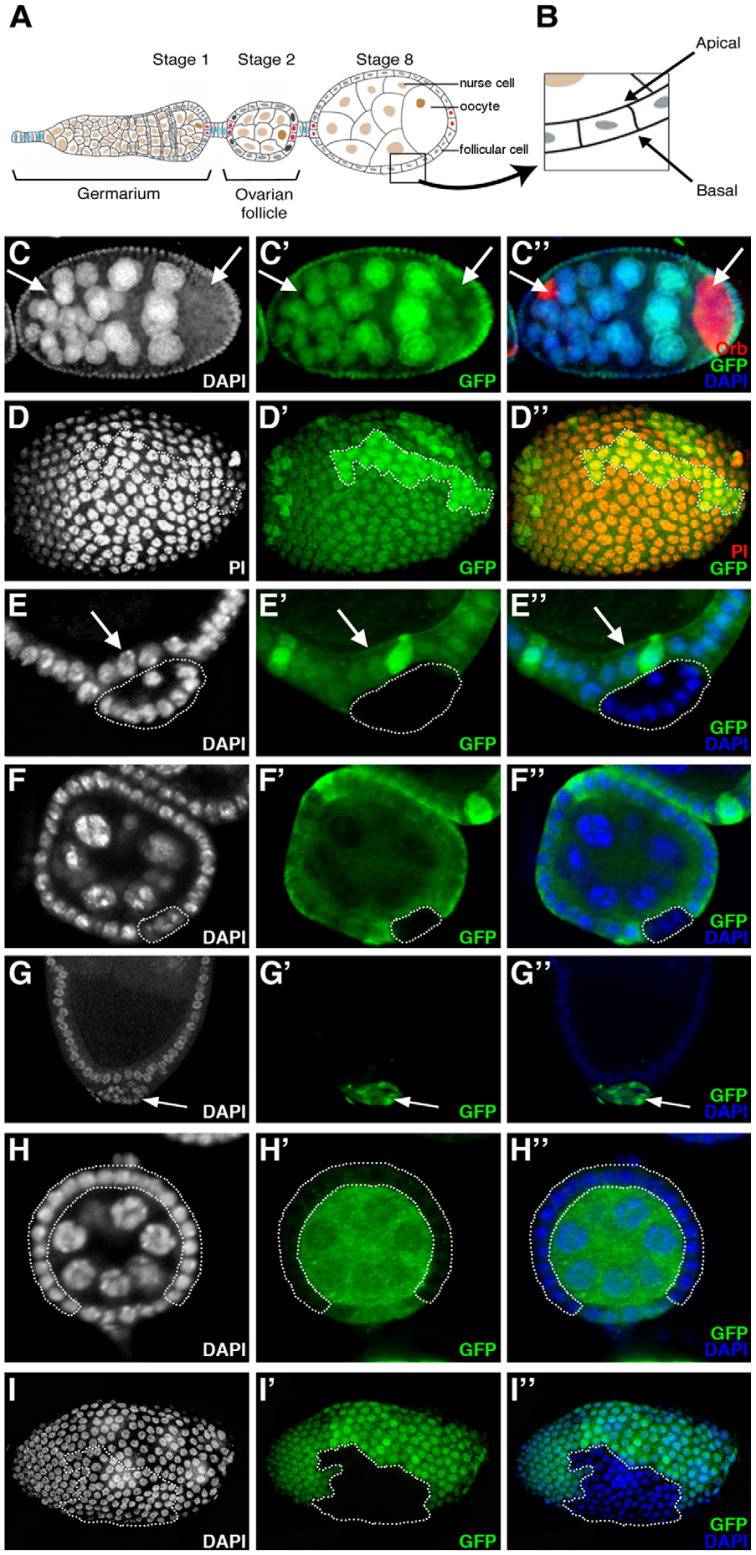
*ph* mutant cells are progressively expulsed from the follicular epithelium in *Drosophila* ovaries. (A) Drawing of a wild-type ovariole (anterior towards left). The stage 2 ovarian follicle buds off from the germarium and matures progressively towards the posterior. Each follicle is composed of a germline cyst of 16 cells, one oocyte (brown) and 15 nurse cells (tan), enveloped by a monolayered epithelium of follicular cells of somatic origin (grey). Specialized somatic cells, polar cells (red), terminal follicle cells (dark blue) and interfollicular stalk cells (light blue), are indicated [61]. (B) Magnified view of the follicular epithelium with the apico-basal orientation indicated. (C–F,H) Follicles in which nuclei are stained with DAPI (C,E,F,H) or Propidium Iodide (PI) (D), *ph* wild-type cells with anti-GFP antibodies (C′,D′,E′,F′,H′) and oocytes with anti-Orb antibodies (C″,arrows). *ph^0^* homozygous clones are detected by the absence of GFP staining. (C–C″) Follicle dissected 8 days after clone induction does not reveal any *ph^0^* cells (C′) but contains more than 15 nurse cells (C) and 2 oocytes (C″,arrows). (D–D″) Surface view of a follicle dissected 8 days after clone induction bearing twin spots (D′, brighter green - encircled and D″, yellow -encircled). (E–E″) *ph^0^* clone (encircled) expulsed from the wild-type follicular epithelium (arrows) of a follicle dissected 5 days after clone induction. (F–F″) In a follicle isolated 2 days after clone induction, a *ph^0^* clone (encircled) is found within the follicular epithelium. (G–G″) Follicle with a clone expressesing GFP (G′,G″,arrow) and an RNAi construct against *ph* which is expulsed from the follicular epithelium (G,G″,arrow). (H–H″) Follicle dissected 5 days after clone induction with a large *ph^0^* follicular cell clone (encircled), which forms a normal follicular epithelium (H,H″). (I–I″) Follicle dissected 8 days after induction of a clone of cells homozygous for *Sce^1^* detected by absence of GFP (I′, encircled) that is correctly integrated in the follicular epithelium (I,I″).

In order to study the function of *ph* in follicular epithelial cells of the *Drosophila* ovary (Figure 1A,B), we used *ph^504^* which is an amorphic allele (noted *ph^0^*) with at least two mutations that completely abolish the function of both the *ph-p* and *ph-d* units of the *ph* locus [13]. To circumvent the embryonic lethality associated with these *ph* mutations, we induced clones of ovarian follicular cells homozygous for the *ph^504^* allele using the mosaic Flp/FRT system [27]. With the Flp/FRT system, mitotic recombination events produce a clone of GFP-negative cells homozygous for the mutation of interest and a corresponding “twin spot” clone identifiable by the presence of two copies of GFP. We first induced clones in females at eclosion and dissected ovaries 8 days later. No *ph^0^* follicular cells (marked by the absence of GFP) were observed under these conditions (Figure 1C′). Nevertheless, the presence of twin spots (marked by high levels of GFP) (Figure 1D′, encircled) indicated that mutant clones were induced, but not recovered. In addition, these ovaries presented phenotypes corresponding to defects during early oogenesis that we have previously reported as being associated with homozygosity for the hypomorphic *ph^lac^* allele and for *ph^0^* mutant cell clones induced in the follicular epithelium [25]. In particular, we observed follicles with an excess number of nurse cells enveloped within a common monolayered follicular epithelium (Figure 1C). These follicles likely result from the encapsulation of two germline cysts together in the germarium as evidenced by the presence of twice the normal number of nurse cells (Figure 1C) and the presence of two oocytes, the latter marked specifically by anti-Orb antibodies (Figure 1C″, arrows). The fact that this mutant phenotype was observed despite the absence of homozygous *ph^0^* follicular cells further supports the hypothesis that *ph^0^* mutant clones were indeed induced, but somehow lost.

We next dissected flies 4–5 days (instead of 8 days) after heat-shock induction of *ph^0^* clones. Indeed, under these conditions, we were able to observe some follicular cells homozygous for *ph^0^* (Figure 1E–E″, encircled), however these *ph* mutant cells were found exterior to the monolayered epithelium composed exclusively of wild-type cells (Figure 1E–E″, arrow). Finally, when females were dissected 1 to 2 days after clone induction, some *ph^0^* homozygous cells were recovered within the follicular epithelium (Figure 1F–F″, encircled). Therefore, by dissecting ovaries at different times after clone induction, we observed that clones of follicular cells mutant for *ph* are progressively lost because they are expulsed from the follicular epithelium. Wild-type follicular cells form a monolayer between the expulsed *ph* mutant cells and the germ cells thereby assuring the continuity of the follicular epithelium.

We also used a transgenic RNAi approach to abolish *ph* function in a restricted number of cells in the ovarian follicular epithelium. We induced clones of cells that express an RNAi construct directed against both *ph-p* and *ph-d* and presenting no off-targets (line 50027: Vienna Drosophila RNAi Center) using the Flp-out technique and the UAS/GAL4 system (see Materials and Methods). The *ph* RNAi-expressing clones, visualized by GFP expression (Figure 1G′,G″, arrow), were also found exterior to the follicular epithelium composed of wild-type GFP^−^ cells (Figure 1G–G″). Therefore, using two different genetic tools to knock down *ph* function, we obtained the same expulsion phenotype, confirming that it is the specific loss of function of *ph* that is responsible for the phenotype. Also, using both approaches, only *ph* mutant cells are expulsed, indicating the cell autonomous nature of the phenotype.

In some cases, we obtained ovarian follicles where the majority of somatic cells are mutant for *ph* (Figure 1H′, encircled) and the follicular epithelium seems perfectly normal as evidenced by the regular organization of the nuclei of the mutant cells (Figure 1H,H″, encircled). These results indicate that cells devoid of *ph* function are still able to ensure their role as epithelial cells in the context of a large clone. Therefore, expulsion of small *ph^0^* mutant clones may be specifically due to the juxtaposition of *ph* wild-type and mutant cells, while a large population of *ph* mutant cells seems to be able to maintain epithelial stability. For the rest of this study, we concentrated on the characterization of the expulsion phenotype of the small *ph* mutant clones.

We next considered whether *ph^0^* mutant cells are dying in which case extrusion might reflect a response to cell death. To test this hypothesis, we used the TUNEL assay, which reveals DNA fragmentation characteristic of cell death by apoptosis. We did not see any TUNEL staining in cells mutant for *ph* (data not shown) indicating that extrusion is not simply a secondary consequence of cell death. In addition, DNA staining with DAPI confirms normal nuclear morphology within the extruded clones (see Figure 1E, encircled, for an example).

Since *ph* is a member of the *PcG* genes, we tested whether mutations in other *PcG* genes lead to similar phenotypes. Using clonal analysis of amorphic mutations, no extrusion of follicular epithelial cells mutant for *Asx*, *Pcl*, *Sce*, *Scm* or doubly mutant for *Psc and Su(z)2*, was observed. We recovered mutant clones correctly integrated within the follicular epithelium and these were of the same size and frequency as twin spot control clones (see *Sce^1^* mutant clones as an example in Figure 1I–I″, encircled and data not shown). In addition, trans heterozygous genetic interactions were tested between mutations in *ph* and other *PcG* genes and no effects on oogenesis phenotypes were observed (data not shown and [25]).

### Expulsion of *ph* mutant cells in the ovarian epithelium is accompanied by a loss in cell polarity

**Figure 2:**
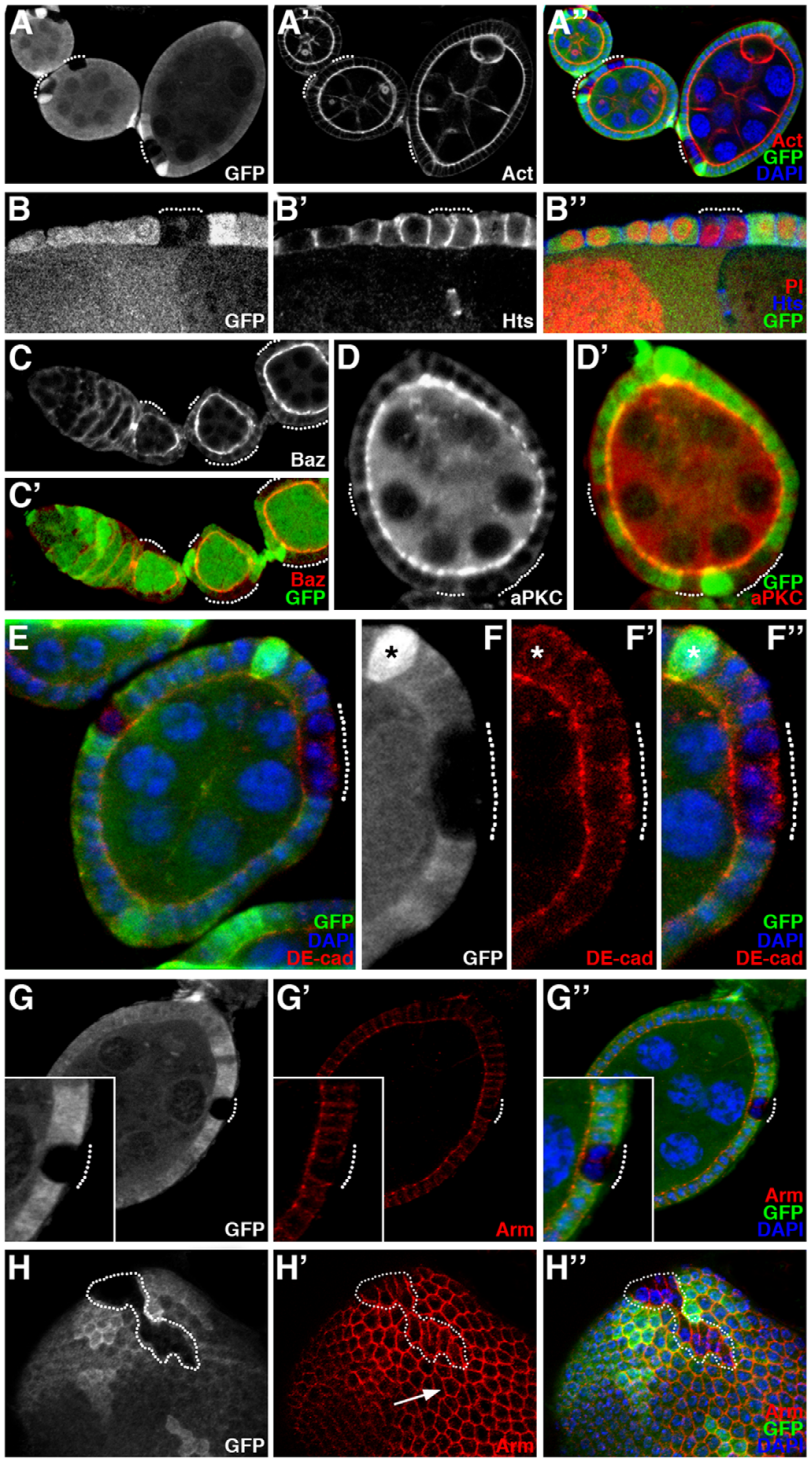
*ph* mutant cells within the follicular epithelium exhibit morphological and adhesion molecule modifications. Analysis of mosaic ovarian follicles with *ph^0^* mutant follicular cell clones integrated within the follicular epithelium (identified by the absence of GFP), using immunodetection to assay for distribution of cytoskeletal markers such as F-actin (Act) and Hu-li tai chao (Hts), apical markers such as Bazooka/Par3 (Baz) and aPKC, and adherens junctions components DE-cadherin (DE-cad) and Armadillo/β-catenin (Arm). All nuclei are stained with DAPI. (A–B) Apical accumulation of F-actin (A′) and lateral distribution of Hts (B′) are normal in the *ph^0^* mutant clones (dotted lines). (C–D) The proteins Baz (C) and aPKC (D) are also correctly localized at the apical membrane in cells mutant for *ph* (dotted lines). (E) The distribution of the apical transmembrane protein DE-cadherin is affected in *ph^0^* mutant clones (dotted lines) since significant amounts of basal accumulation are observed (F–F″, magnification of the clone in E). In F–F″, the asterisk indicates one of the polar cells which also exhibits some basal DE-Cadherin. (G–G″) Apical Armadillo/β-catenin distribution is not significantly disturbed in *ph^0^* mutant cells (dotted lines). (H–H″) Adherens junction organization between *ph* wild-type epithelial cells produces a hexagonal honeycomb pattern of Armadillo/β-catenin staining in a surface view of a follicle (H′-arrow), while detection of this protein in clones of cells mutant for *ph* (H–H″ GFP negative, encircled) reveals cell morphological modifications including distinct elongation and apical constriction.

One explanation for the segregation of *ph^0^* and *ph^+^* epithelial follicular cells could be differential cell adhesion between the two cell populations. We therefore next examined the expression of several proteins implicated in cell-cell adhesion and/or polarization of epithelial cells. Several apical markers (F-actin, Bazooka/Par3, aPKC, Crumbs), a lateral marker (Hu-li tai shao, Hts) and basal markers (Integrin-βPS and Talin) were tested. In *ph^0^* mutant follicular cells that are still integrated in the follicular epithelium, F-actin (Figure 2A–A″, dotted lines), Bazooka/Par3 (Figure 2C,C′, dotted lines), aPKC (Figure 2D–D′, dotted lines) and Crumbs (data not shown) were expressed at wild-type levels and maintained their apical subcellular localization. Hts (Figure 2B–B″, dotted line), Integrin-βPS and Talin (data not shown) were not disturbed in *ph^0^* mutant cells that are still integrated in the follicular epithelium. However, delocalization of part of the DE-cadherin pool, one of the components of apical adherens junctions, towards the basal/lateral membrane was observed in *ph^0^* mutant cells (Figure 2E,F–F″, dotted line), though distribution of a second adherens junction component, Armadillo/β-catenin, was not significantly affected (Figure 2G–G″, dotted line). Surface views of mosaic follicles also showed a difference between *ph^+^* and *ph^0^* cells. In these views, when the focal plain was placed at the apical side of follicular epithelial cells, *ph^+^* cells display a regular hexagonal ring pattern of staining for adherens junction components (for Armadillo/β-catenin, Figure H′, arrow and data not shown). In contrast, *ph^0^* cells were deformed, in particular exhibiting significant elongation that increased contact between adjacent *ph^0^* cells and decreased contact between *ph^0^* and *ph^+^* cells (Figure 2H–H″, encircled). At this stage, the *ph^0^* mutant cells are still integrated in the follicular epithelium, but they have undergone cell shape changes that indicate segregation from the rest of the *ph^+^* epithelial cells. These modifications in morphology and thus in interaction with neighboring cells, as well as delocalization of DE-cadherin towards the basal/lateral membrane, therefore precede expulsion of *ph^0^* mutant cells and likely participate actively to the process.

**Figure 3:**
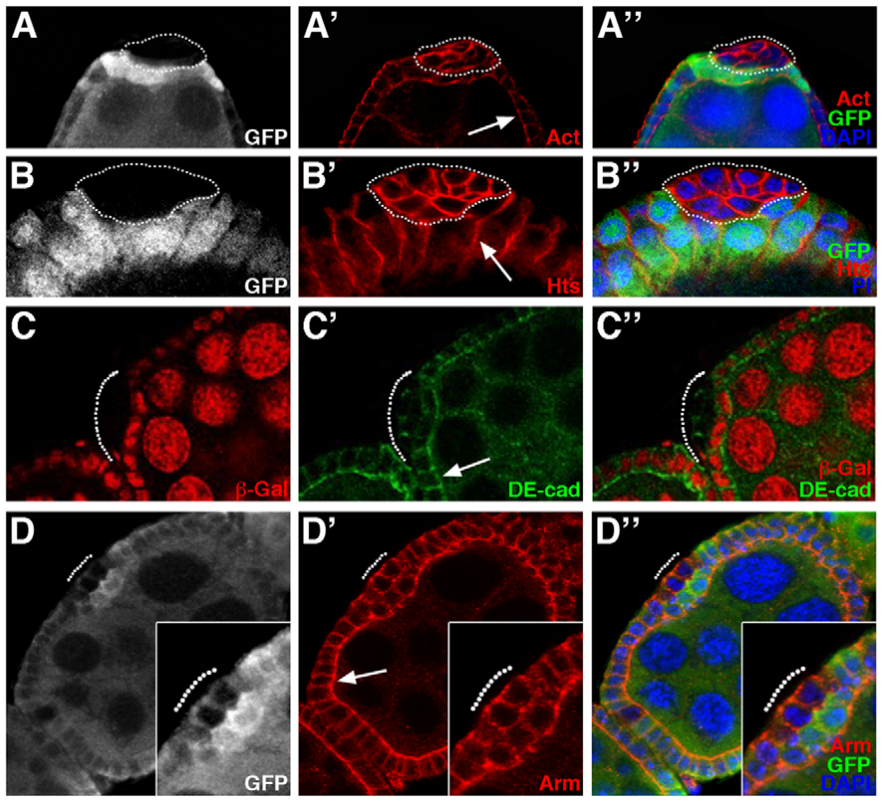
Adhesion and cytoskeletal protein distribution is modified in *ph^0^* cells expulsed from the follicular epithelium. Mosaic ovarian follicles with *ph^0^* mutant clones identified by the absence of GFP (A, B, D) or β-galactosidase (β-Gal) staining (C). Nuclei are marked by DNA staining with DAPI (A″, D″) or Propidium Iodide (PI) (B″). Using immunodetection, we examined the distribution of the cytoskeletal markers F-actin (Act) and Hu-li tai chao (Hts), and of the adherens junction components DE-cadherin (DE-cad) and Armadillo/β-catenin (Arm) in cells mutant for *ph* that are expulsed from the follicular epithelium (indicated by dotted lines), compared to that in neighboring wild-type cells (white arrows). (A–B) F-actin (A′) and Hts (B′) are localized cortically in the *ph^0^* mutant clones, which adopt a round shape and lose their monolayer epithelial organization. (C) In a clone mutant for *ph* (C,C″), DE-cadherin is localized at the membranes between *ph* mutant cells (C′,C″). (D) A *ph^0^* mutant clone extruded from the follicular epithelium (D,D″) exhibits cortical staining for Armadillo/β-catenin (D′,D″).

We performed the same type of analysis on *ph^0^* mutant cell clones already expulsed from the regular follicular epithelium. In wild-type cuboidal ovarian follicular cells, F-actin is preferentially localized apically (Figure 3A′, arrow) and Hts laterally (Figure 3B′, arrow). When *ph^0^* mutant cells were expulsed as a small cell mass (Figure 3A–A″,B–B″, encircled), they adopted a more round, irregular shape, and F-actin and Hts accumulated at high levels at the interface between mutant cells. The basal markers Integrin-βPS and Talin were also localized throughout the membrane in the expulsed *ph^0^* mutant cells (data not shown). We also examined DE-cadherin and Armadillo/β-catenin, which are normally at their highest levels apically in wild-type epithelial follicular cells (Figure 3C′,D′, arrows). In *ph^0^* cells that are expulsed, DE-cadherin was present only at the interface between *ph^0^* mutant cells (Figure 3C′–C″, dotted line) and Armadillo/β-catenin was localized cortically (Figure 3D′–D″, dotted line). Therefore, *ph* mutant follicular cells show loss of apico-basal polarity once they are expulsed. Importantly, wild-type cells were always able to organize themselves such that a new monolayered epithelium was formed beneath the expulsed *ph^0^* mutant cells (Figure 3A″,B″,C″,D″). Taken together, these results suggest that remodeling of adherens junction organization between *ph* mutant cells and wild-type cells, such that new connections are preferentially formed between “like” cells, leads to segregation of the *ph* mutant clones from the follicular epithelium.

### *ph* mutant cells in the wing imaginal disc epithelium are extruded basally as a cyst-like structure with modified apical and basal domains

**Figure 4:**
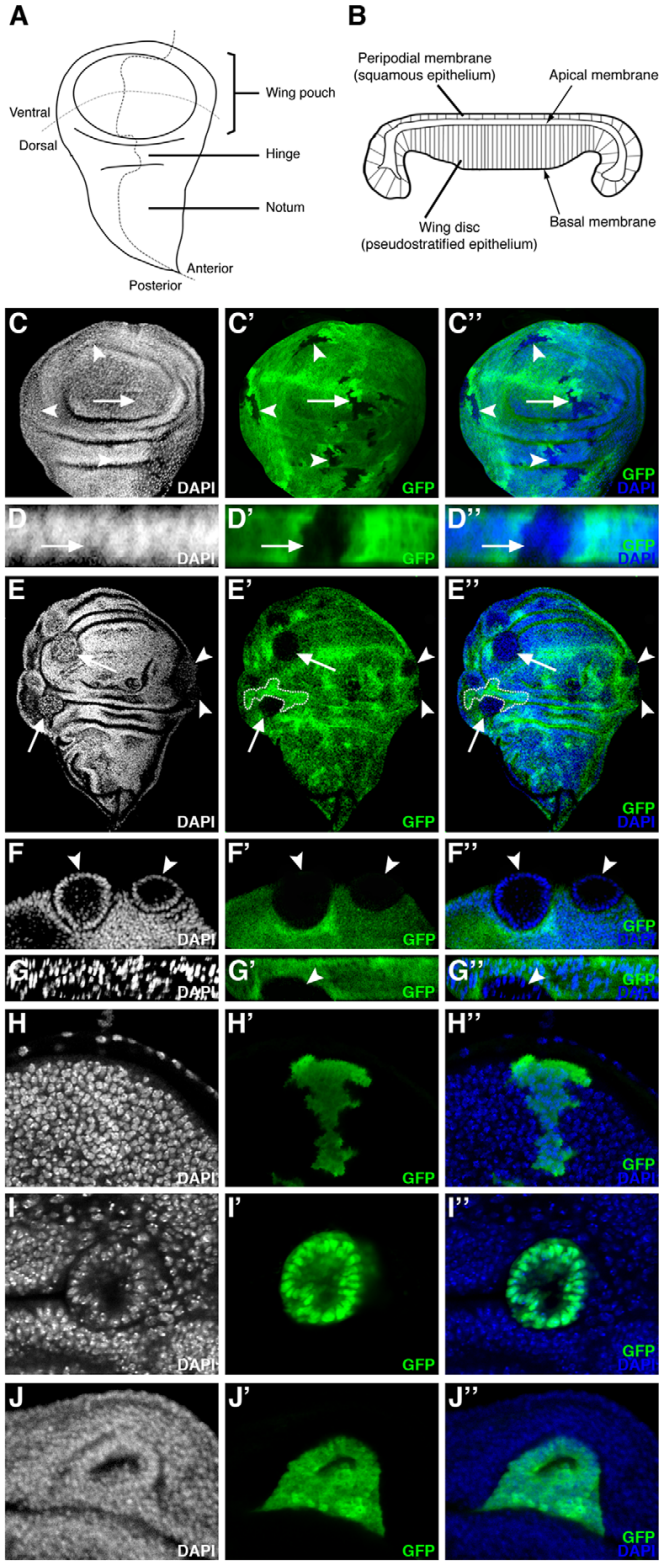
In the wing imaginal disc, *ph* misexpression clones sort-out from the wild-type epithelium. (A) Drawing of a wing imaginal disc with compartments and presumptive territories indicated. (B) Drawing of a cross-section of a wing imaginal disc at the level of the wing pouch [47]. (C–G) Wing discs in which clones of cells have been induced which lack GFP (C–D) or lack both GFP and *ph* function (E–G). Panels C, E and F are XY confocal sections (orientation as in A). (C–C″) Wild-type clones lacking GFP form clones with wiggly borders evenly integrated in the epithelium (arrow and arrowheads). (D–D″) A XZ cross-section of the wing pouch (orientation as in B) of a wild-type clone (C–C″-arrow) showing GFP-negative cells integrated normally into the epithelial layer. (E–E″) *ph^0^* homozygous clones lacking GFP segregate from surrounding control cells, disrupting the continuity of the epithelial layer and forming round clones with smooth borders (arrow and arrowheads). A twin clone with wiggly borders, indicated with dotted lines, is of approximately the same size as what is likely its corresponding *ph^0^* clone. (F–F″) Magnified view of two mutant clones (arrowheads) from E (arrowheads), showing that *ph^0^* cells are organized as monolayered spheres which bud off the wing disc. (G–G″) XZ cross-section (orientation as in B) of a *ph^0^* clone (arrowhead) extruded basally as a cyst. (H,I,J) XY confocal views of wing discs in which clones of cells expressing GFP form wiggly borders with adjacent non-GFP expressing cells and are integrated normally in the disc epithelium (H), whereas clones expressing both GFP and the *ph* RNAi construct (I,) or GFP and the *ph* overexpression construct (J) form smooth borders with neighboring wild-type cells and a cyst structure. DAPI marks all nuclei.

Our finding that *ph^0^* cells are expulsed from the ovarian follicular epithelium was intriguing since previous studies indicated that induction of *ph^0^* clones in the wing imaginal disc also leads to a poorly-characterized expulsion phenomenon which is associated with the presence of cuticular vesicles lodged between the ventral and the dorsal surfaces of the adult wing (or in the legs) [22], [24]. The wing imaginal disc, the wing primordium present in the larva, is another important model system for the study of epithelial organization in *Drosophila* (Figure 4A,B). In order to further characterize this phenomenon, we induced wild-type and *ph^0^* clones in first instar larvae using a heat-shock inducible source of flipase and dissected wing imaginal discs of third instar larvae (see Materials and Methods). Wild-type clones (marked by the absence of GFP) induced in all regions of the wing disc formed wiggly borders with their neighboring non-clonal cells (Figure 4C′,C″, see arrow and arrowheads for examples). Wild-type clones formed an integral part of the epithelial layer as evidenced by the regular distribution of the nuclei in both X-Y views of the wing disc (Figure 4C, arrow/arrowheads) and Z optical cross sections (Figure 4D, D″, arrow). In contrast, we found that *ph^0^* mutant clones adopted a round shape and segregated from surrounding wild-type cells forming smooth borders with their neighboring cells (Figure 4E–E″, arrows/arrowheads). Indeed, the cells in *ph^0^* mutant clones reorganized and formed a sphere of only one layer of cells as evidenced by the organization of the nuclei revealed by DAPI staining in cross sections of the clones taken from both X/Y (Figure 4F–F″, arrowheads) and X/Z axes (Figure 4G–G″, arrowhead). Visualized in Z optical cross sections, cells mutant for the *ph* gene were not maintained in the pseudostratified layer of epithelial cells, resulting in their retraction from the basal epithelial surface and subsequent expulsion (Figure 4G–G″, arrowhead). We routinely observed expulsion of *ph^0^* clones in the wing pouch, as well as in the presumptive hinge and notum imaginal tissue (Figure 4E–E″, arrowheads/arrows and data not shown). In addition, we did not observe any significant difference between the size and frequency of *ph^0^* mutant and wild-type clones induced independently (compare Figure 4C′ and E′ and data not shown) or between *ph^0^* mutant and wild-type twin clones (Figure 4E–E″, dotted lines indicate a twin clone, and data not shown) indicating that under the conditions used, the expulsion phenotype is not a consequence of proliferation or growth defects. Using these same conditions, similar results were obtained for other imaginal discs including that of the eye-antenna (Figure S1A). If different conditions for heat shock induction of clones were used, such as conducting heat shocks earlier in development, then larger clones exhibiting clear segregation from wild-type tissue were recovered in wing, as well as eye-antennal, imaginal discs (Figure S1D,E and data not shown). Multiplying the number of heat shocks and starting these early in development allowed recovery of rare imaginal discs (eye-antenna, wing and leg) with overgrowth phenotypes (Figure S1F and data not shown), resembling those previously reported [16], [17]. Since we were interested in analyzing the effects of *ph* mutations specifically on epithelial integrity, we continued the analysis using heat shock conditions that allow uncoupling of the epithelial instability and more severe overgrowth phenotypes.

A very similar expulsion phenotype was observed when clones of cells that express an RNAi construct targeting both *ph* units were induced (VDRC 50027 line). As a control, clones of cells expressing GFP ectopically in the wing disc were induced using the Flp-out technique and the UAS/GAL4 system (see Materials and Methods). These GFP^+^ clones formed wiggly borders with neighboring GFP^−^ cells (Figure 4H′,H″) and were integrated normally in the wing disc (Figure 4H). In contrast, expression of both GFP and the *ph* RNAi construct led to production of clones with smooth borders consisting of a monolayer of cells organized as a cyst (Figure 4I–I″). Similar results were obtained for other imaginal discs including that of the eye-antenna (Figure S1B,C). In addition, *ph* RNAi clones also exhibited derepression of the homeotic gene *Abd-B*, like *ph^0^* clones ([22]), further supporting that the *ph* RNAi construct specifically reduces *ph*. Therefore, by two independent methods, loss of function of *ph* is specifically associated with the expulsion phenotype in the wing imaginal disc. Thus, in two different epithelial models, the ovarian follicle and the wing imaginal disc, juxtaposition of *ph* mutant and wild-type cells leads to epithelial instability.

We also tested whether overexpression of *ph* had an effect on epithelial organization in the wing imaginal disc. In contrast to clones of cells expressing only GFP ectopically (Figure 4H–H″), clones which in addition overexpressed *ph* formed smooth borders with neighboring wild-type cells and in some cases reorganized into a cyst structure clearly segregating from the rest of the disc (Figure 4J–J″). Therefore, induction of clones of cells with either reduced or increased *ph* expression both lead to segregation of the clones from neighboring wild-type cells with normal PH levels.

**Figure 5:**
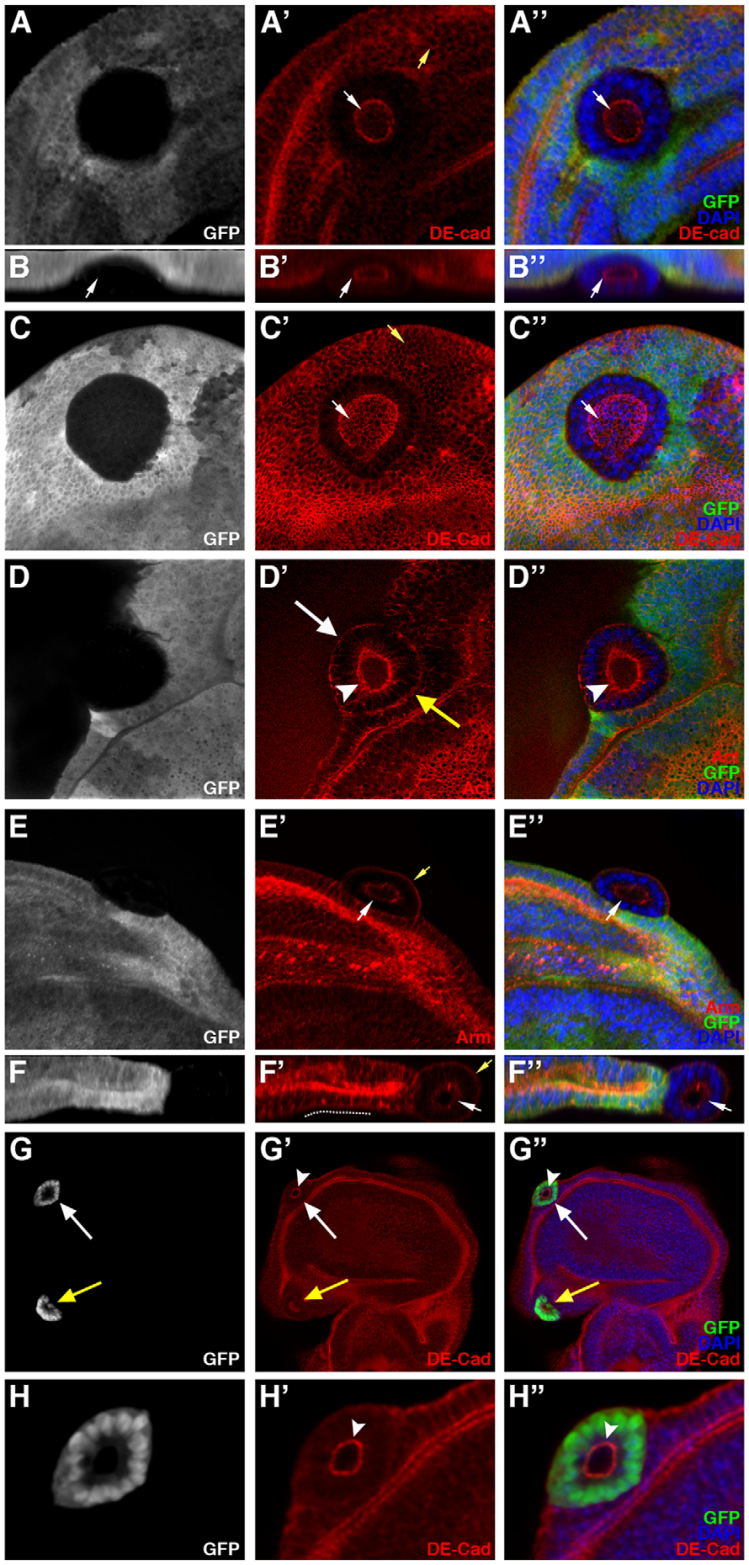
Redistribution of polarity and adhesion markers in *ph^0^* wing imaginal disc clones expulsed basally. (A–F) Mosaic wing discs in which the cells mutant for *ph* are identified by the absence of GFP, DAPI marks all the nuclei and antibodies reveal the distribution of DE-cadherin (DE-cad), F-actin (Act) and Armadillo/β-catenin (Arm). Merged views of the triple stainings are presented in A″–F″. Panels A, C, D and E are standard confocal XY sections (discs are oriented with ventral towards the top). Panels B and F are XZ optical cross sections of the mutant clones presented in panels A and E, respectively (discs are oriented as in Figure 4B). (A, B and C) DE-cadherin immunostaining (red) reveals DE-cadherin localized at the internal face of the *ph* mutant clones (A′,A″,B′,B″,C′,C″-white arrows). The yellow arrow in A′ and C′ shows the apical DE-cadherin staining in wild-type cells. (D) F-actin (red) is localized at the internal (D′,D″, white arrowhead) and the external (D′, white and yellow arrows) surface of the cells mutant for *ph*. (E and F) The apical protein Armadillo/β-catenin (red) is present at the internal membrane of the *ph* mutant cells (E′,E″,F′,F″-white arrows) and also externally towards the surrounding wild-type cells (E′,F′- yellow arrow). (G) Wing disc (oriented with ventral towards the top) bearing clones of cells expressing an RNAi construct targeting *ph* marked by the presence of GFP (G,G″- white and yellow arrows). All nuclei are marked with DAPI (G″). DE-cadherin (DE-cad) is revealed by immunodetection (G′,G″). Panel H is a magnified view of the mutant clone marked by a white arrow in panel G. Clones of cells that express the *ph* RNAi construct are organized as a monolayered sphere with smooth borders (G,G″,H,H″). DE-cadherin is localized in the center of the clones as a ring (G′,G″,H′,H″, white arrowheads).

In order to characterize more precisely the reorganization of *ph^0^* mutant cells during expulsion in the wing disc, we examined the expression of markers implicated in the polarization of epithelial cells. In wild-type wing disc cells, DE-cadherin forms apical cell-cell junctions with all immediate neighboring cells, thereby generating an apical ring, close to hexagonal in shape, at the cellular level and an apical honeycomb-like lattice at the tissular level (Figure 5A′,C′, yellow arrows). In clones of *ph^0^* cells in the wing disc, DE-cadherin was localized exclusively between *ph* mutant cells towards the center (internal face) of the clone (Figure 5A′,A″, white arrow). Indeed, in all optical cross sections of the same *ph* mutant clone, including along the Z axis, DE-cadherin staining takes the form of a ring present in the center of the clone (Figure 5B′,B″, arrow). Upon visualization of DE-cadherin on the internal face of the *ph^0^* mutant clone with a surface, rather than transverse view, a honeycomb lattice cage-like spherical structure was observed (Figure 5C′,C″ white arrow) resembling the organization of adherens junctions between wild-type cells in a normal epithelium (Figure 5A′,C′ yellow arrows). The *ph* RNAi clones which are also extruded as a cyst-structure from the wing disc epithelium (Figure 5G–G″, arrows) exhibited the same organization of DE-Cadherin between the mutant cells towards the center of the clone (Figure 5H–H″, arrowhead).

We also examined the accumulation of two other apical markers, F-actin and Armadillo/β-catenin. F-actin (Figure 5D′,D″, arrowhead) and Armadillo/β-catenin (Figure 5E′,E″ and 5F′,F″, white arrows), like DE-cadherin, were localized at the internal face of *ph* mutant clones. However, unlike DE-cadherin, these two proteins were also present at the external face of *ph^0^* cell clones towards the exterior of the disc (Figure 5D′, white arrow and E′, yellow arrow) or towards *ph^+^* cells (Figure 5D′, yellow arrow). Therefore, *ph* mutant cells extruded as a cyst establish new apical contacts with each other composed of adherens junction components and F-actin, but the opposite pole of these cells may not fully resemble a normal basal domain since there is some accumulation of Armadillo/β-catenin and F-Actin.

**Figure 6:**
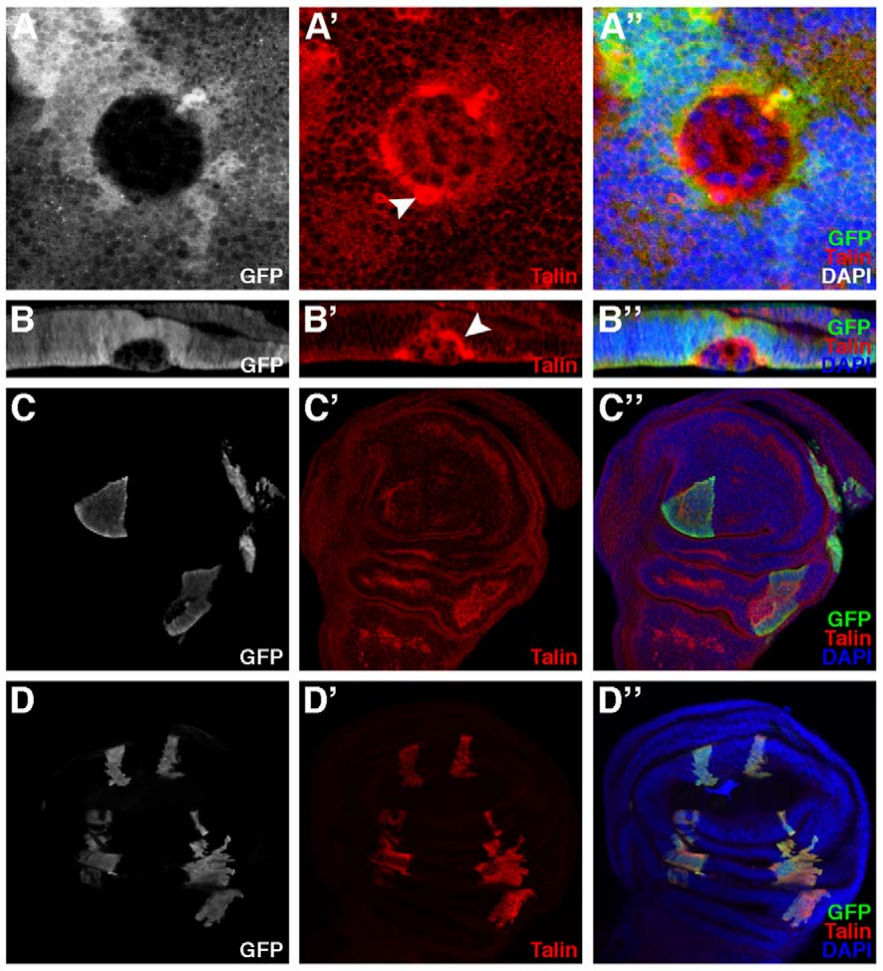
Clonal loss of function and overexpression of *ph* and *rhea* in the wing imaginal disc. (A, B) Mosaic wing disc in which cells mutant for *ph* are identified by the absence of GFP, DAPI marks all the nuclei and the distribution of the basal marker Talin is revealed by immunodetection. Merged views of the triple stainings are presented in A″–D″. Panel A is a standard confocal XY section (disc is oriented with ventral towards the top). Panel B is a XZ optical cross section of the *ph* mutant clone observed in A (disc is oriented as in Figure 4B). In *ph^0^* mutant clones, an abnormally high level of Talin (red) accumulates at the surface in contact with the wild-type surrounding cells (A′,A″,B′,B″-white arrowheads). (C) Mosaic wing disc (oriented with ventral towards the top) bearing clones of cells that express GFP ectopically (C,C″) along with an overexpression *ph* construct. All nuclei are stained with DAPI (C″) and Talin accumulation is revealed with specific antibodies (C′,C″). Clones of cells that overexpress *ph* form smooth borders with the wild-type surrounding cells (C,C″) but Talin accumulation is similar between the *ph* overexpressing cells and surrounding wild-type cells (C′,C″). (D) Mosaic wing disc (oriented with ventral towards the top) bearing clones of cells that express GFP ectopically (Figure D,D″) as well as a *rhea* overexpression construct. High level Talin accumulation specifically in the clones is revealed with specific antibodies and exposure settings that do not allow visualization of the lower endogenous level of Talin in non-clonal cells (D′,D″). All nuclei are stained with DAPI (D″). The cells that accumulate elevated levels of Talin form clones with heterogeneous morphology and wiggly borders which do not sort-out from the wild-type cells (D,D″).

We therefore next examined the accumulation of the basal marker Talin in *ph* mutant clones in the wing imaginal disc. A much greater amount of Talin was observed in *ph* mutant cells compared to adjacent wild-type cells, some present as diffuse staining in the cytoplasm, but more significantly as large patches of staining towards the external face of the clones next to *ph^+^* cells (Figure 6A′,B′, arrowhead). Taken together, these results indicate that the expulsed cyst-like structures are comprised of *ph^0^* cells that are repolarized such that a new apical domain faces the center of the clone and an abnormal basal domain, exhibiting high levels of Talin accumulation, forms towards the exterior of the clone.

In order to further explore the relationship between Talin accumulation and the expulsion phenotype associated with *ph* mutations, we first tested whether overexpression of *ph* had any effect on Talin accumulation. We induced clones of cells expressing GFP and overexpressing *ph* in the wing disc using the Flp-out technique and the UAS/GAL4 system (see Materials and Methods). Although these clones, marked by GFP expression, exhibited smooth borders with neighboring wild-type cells (Figure 6C,C″), they did not exhibit altered accumulation of Talin (Figure 6C′,C″). Next, we tested whether direct overexpression of Talin, encoded by the *rhea* gene, had any effect on epithelial stability. Clones overexpressing Talin, as visualized by Talin-specific antibodies (Figure 6D′) and GFP expression (Figure 6D, D″), were indistinguishable from wild-type clones (see Figure 4H″) in that they formed wiggly borders and were normally integrated in the wing disc epithelium (Figure 6D–D″). These results indicate that an abnormally high accumulation of Talin is not sufficient to provoke sorting out of a clonal wing disc cell population.

### Some *ph^0^* mutant clones in the wing disc are also expulsed apically with complete loss of cell polarity

**Figure 7:**
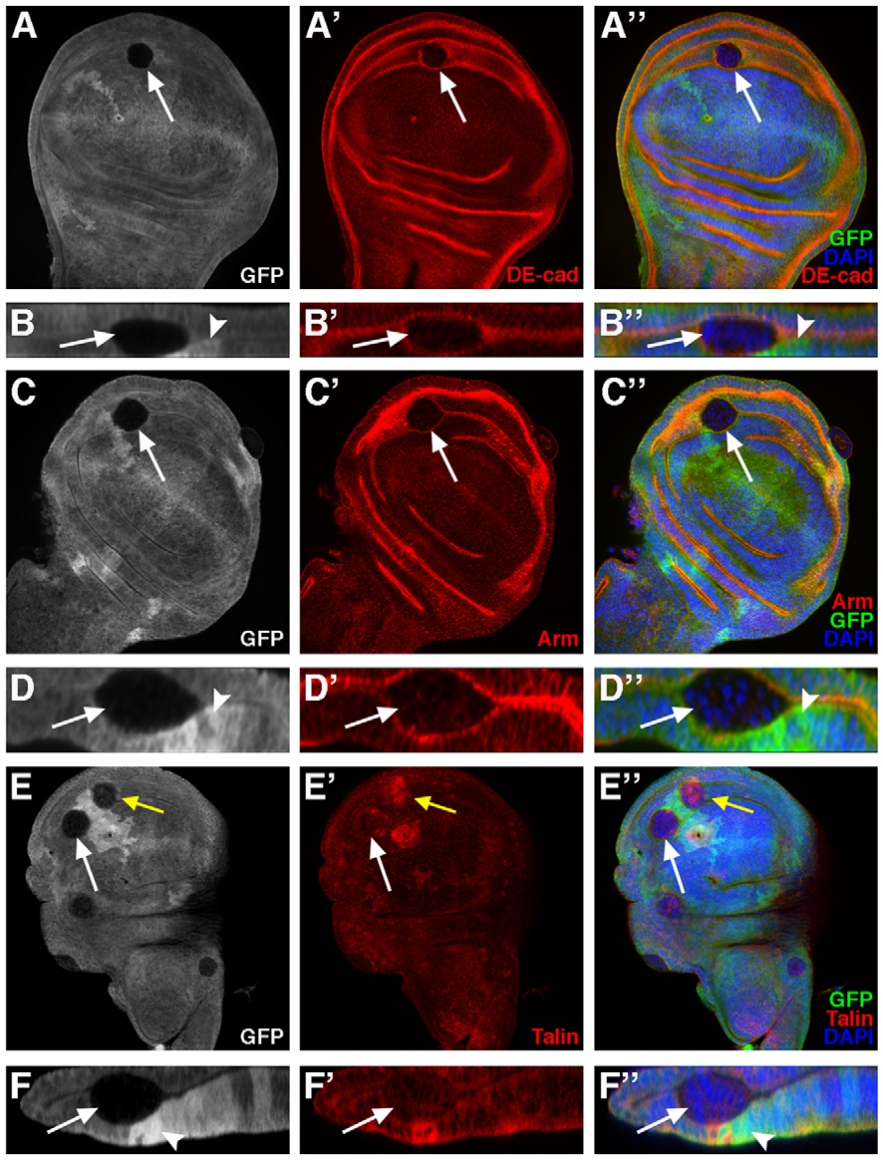
In the wing imaginal disc, *ph^0^* cells expulsed apically display complete loss of apico-basal polarity. Mosaic wing discs in which cells mutant for *ph* are identified by the absence of GFP, DAPI marks all the nuclei and the distribution of DE-cadherin (DE-cad), Armadillo/β-catenin (Arm) and Talin is revealed by immunodetection. Panels A, C and E are standard confocal XY sections (discs are oriented with ventral towards the top). Panels B, D and F are XZ optical cross sections of the mutant clones indicated by white arrows in A, C and E respectively (discs are oriented as in Figure 4B). *ph^0^* mutant clones segregate from the wild-type neighboring cells and adopt a round shape, but the cells exhibit no particular organization (A″,C″,E″, white arrows, as revealed by DAPI-marked nuclei in blue). XZ optical cross sections of the *ph^0^* mutant clones indicate that they are expulsed apically and are sandwiched between the peripodial membrane and the wing disc epithelium (B″,D″,F″, white arrows). DE-cadherin (A′,B′), Armadillo/β-catenin (C′,D′) and Talin (E′,F′) are present diffusely in the cytoplasm of *ph* mutant cells expulsed apically. In B,B″, D,D″ and F,F″, white arrowheads indicate the position of twin spots marked by a double dose of GFP. (E) For comparison, a *ph^0^* mutant clone expulsed basally accumulates abnormally high levels of Talin (E–E″, yellow arrow).

During the study of the extrusion of *ph* mutant cells in the wing disc, we found that some *ph^0^* mutant clones (Figure 7A,C,E, white arrows) were expulsed apically rather than basally and were thus found wedged between the peripodial membrane and the wing disc epithelium as evidenced in the Z cross sections of these clones (Figure 7B,D,F, arrows). The disc, rather than peripodial membrane, origin of these clones was demonstrated by the systematic presence of strongly GFP-positive twin clones in the disc epithelium as observed in Z cross sections (Figure 7B,D,F, arrowheads) and the absence of such twin clones in the peripodial membrane (Figure 7B,D,F). These apically-expulsed *ph^0^* mutant clones, in contrast to the basally-expulsed clones that form a regular cyst structure, exhibited no specific organization of mutant cells as evidenced by DAPI nuclear staining (Figure 7A″–F″, white arrows). In addition, the normally apical adherens junction components, DE-cadherin (Figure 7A′,B′, arrows) and Armadillo (Figure 7C′,D′, arrows), were not polarized, but rather diffuse in the cytoplasm of these cells giving the impression of somewhat reduced accumulation of these proteins. In order to determine whether *ph* mutant clones exhibited downregulation of expression of either the *shotgun* (encoding DE-Cadherin) or *armadillo/β-catenin* genes, *lacZ* transcription reporter constructs were used and no significant difference in the expression levels of these reporters was observed between *ph* mutant and nearby wild-type cells (data not shown). It is therefore possible that the low signal observed upon immunodetection of DE-Cadherin and Armadillo/*β-catenin* in *ph* mutant apically-expulsed clones is merely a consequence of diffuse localization of these proteins. The normally basal protein, Talin, also appeared distributed in a diffuse manner in the cytoplasm of *ph* mutant cells in apically-expulsed clones (Figure 7E′,F′, white arrows) in sharp contrast to the high level of Talin accumulation in basally-expulsed clones present in the same disc (7E′, yellow arrow). Therefore, expulsion of *ph^0^* mutant cells from the wing imaginal disc epithelium can proceed via two dramatically different modes, namely, basal expulsion of a cyst-like structure with apico-basal organization and apical expulsion of a small mass of cells with no apico-basal organization.

### *Abd-B*, a known PH target in the embryo and wing imaginal disc, is also a PH target in the ovarian follicular epithelia

**Figure 8:**
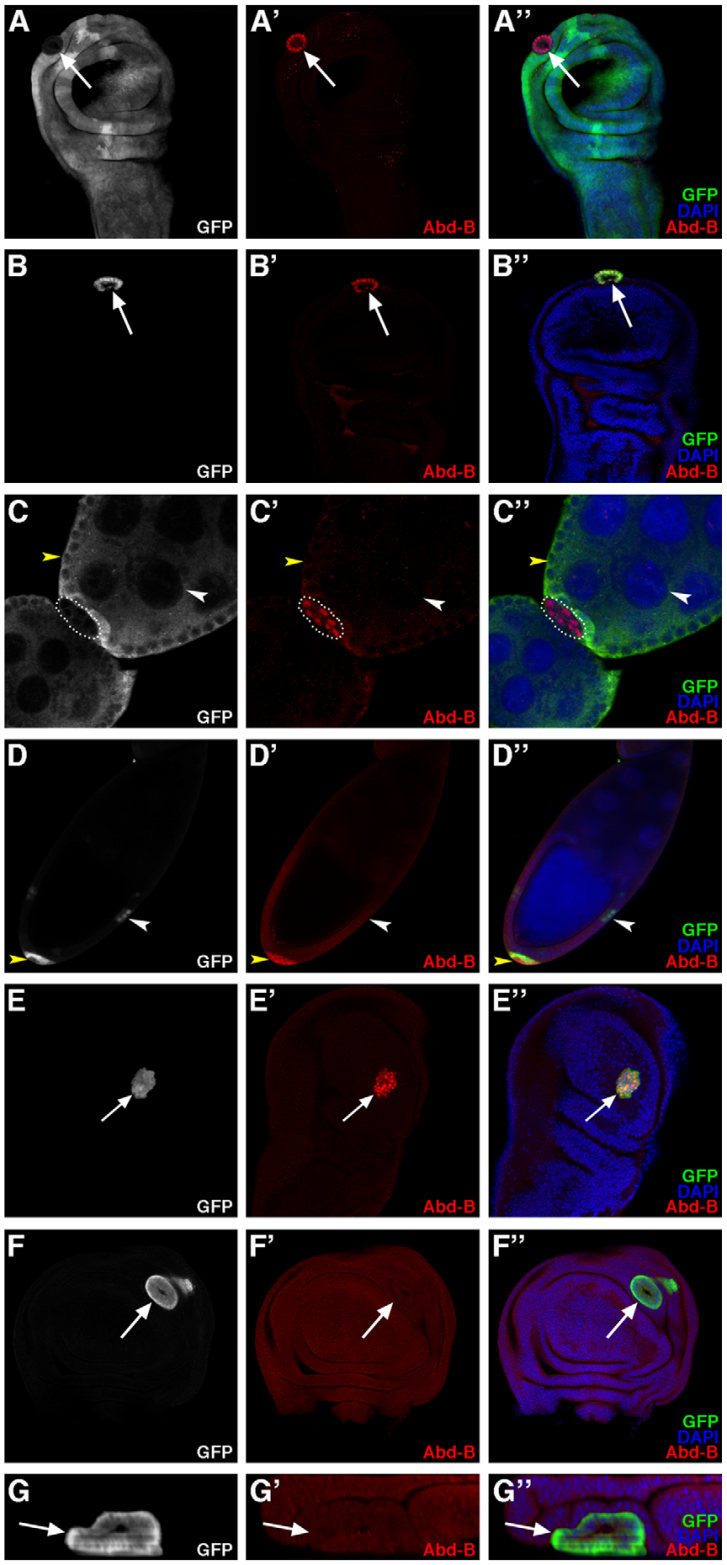
Interaction between *ph* and *Abd-B* in the ovarian follicle and wing imaginal disc. XY confocal sections of wing imaginal discs (A,B,E–F - oriented as in 4A) and ovarian follicles (C,D) revealing Abd-B protein accumulation by immunodetection. All nuclei are marked with DAPI. (A–A″) Wing disc in which a *ph^0^* homozygous cell clone is identified by the absence of GFP (arrow) and Abd-B, not present in surrounding wild-type cells, is accumulates ectopically in *ph* mutant cells (arrow). (B–B″) Wing disc bearing a clone of cells expressing GFP (arrow) along with an RNAi construct targeting *ph*. The clone exhibits a round shape and ectopic expression of *Abd-B*. (C–C″) Ovarian follicular epithelium in which a *ph^0^* mutant cell clone is identified by the absence of GFP (encircled), and Abd-B, not present in the surrounding wild-type follicular and nurse cells, is specifically expressed in the *ph^0^* mutant cells. (D–D″) Ovarian follicular cell clones expressing GFP ectopically (arrowheads) along with an RNAi construct targeting *ph*. A clone of cells expressing the *ph* RNAi construct accumulates Abd-B and is expulsed (yellow arrowhead), whereas another clone that does not accumulate Abd-B is correctly integrated in the follicular epithelium (white arrowhead). (E–E″) Wing bearing a clone of cells that expresses both GFP and *Abd-B* (arrow) and exhibits smooth borders with neighboring wild-type cells. (F–F″) Wing disc bearing a clone of cells that expresses GFP (arrow) along with RNAi constructs targeting *ph* and *Abd-B*. Efficient downregulation of *Abd-B* is obtained with the RNAi construct as revealed by the absence of Abd-B accumulation (arrow). The clone presents smooth borders with neighboring wild-type cells and is expulsed basally from the disc epithelium (G - XZ optical cross section of the clone in F oriented as in Figure 4B).

*Abd-B* is a homeotic gene, which functions in the specification of the posterior abdomen and the genitalia of the fly [28]. In the embryo, *Abd-B* is expressed in the posterior-most parasegments, 10 through 15, and is repressed by PcG products including PH outside of this expression domain [26]. In particular, *Abd-B* is repressed in the wing imaginal disc throughout larval stages and it is derepressed upon induction of *ph* mutant clones in this tissue in a cell autonomous manner as evidenced by the presence of Abd-B protein in nuclei of mutant cells undergoing sorting-out [22] (Figure 8A–A″, arrow). Clonal expression of an RNAi construct targeting *ph* transcripts also led to cell autonomous derepression of *Abd-B* and accumulation of this nuclear protein (Figure 8B–B″, arrow). These observations further confirm that clones of cells expressing the *ph* RNAi construct exhibit the same phenotype as *ph^0^* mutant cell clones (compare Figures 8A–A″ and B–B″).

We examined Abd-B protein accumulation in the adult ovary and found that it was not expressed in either somatic (Figure 8C′, yellow arrowhead) or germline (Figure 8D′, white arrowhead) wild-type cells. In contrast, *Abd-B* was expressed ectopically in nuclei of expulsed *ph* mutant follicular cells using either the *ph^0^* allele (Figure 8C–C″, encircled) or the transgenic RNAi construct targeting *ph* (Figure 8D–D″, yellow arrowhead). We noted however that when using the *ph* RNAi construct, small-sized clones did not exhibit expulsion and did not express Abd-B ectopically (Figure 8D–D″, white arrowhead). Indeed, similar results correlating *ph* mutant clone size and *Abd-B* derepression were reported previously in the wing imaginal disc [22]. Therefore, PH represses *Abd-B* expression in the wild-type adult somatic ovary(as it does in the wing imaginal disc.

### Overexpression of *Abd-B* induces sorting out of cells, but *Abd-B* is not necessary for expulsion of *ph* mutant cells

We next studied the effects of directly overexpressing *Abd-B* in epithelial cells using the Flp-out technique and the UAS/GAL4 system (see Materials and Methods). In the ovarian follicular epithelium, overepression of *Abd-B* lead to several defects in ovary morphogenesis preventing analysis of epithelial phenotypes (data not shown). In the wing disc, recovery of clones of cells that overexpress *Abd-B* and GFP (Figure 8E–E″, arrow) was largely inefficient compared to that of GFP-expressing wild-type clones (data not shown). However, all the *Abd-B* overexpression clones recovered were round clones making smooth borders with neighboring wild-type cells (Figure 8E–E″, arrow), in contrast to the wild-type clones that formed wiggly borders with neighboring tissue (Figure 4H–H″). These results suggest that ectopic *Abd-B* expression, at least in the wing disc epithelium, is sufficient to induce segregation between Abd-B^+^ and Abd-B^−^ cells. Therefore, differential expression of *Abd-B* in an epithelial tissue, as for *ph*, also leads to its instability.

We next tested if epistatic relations exist between *ph* and *Abd-B* for epithelial instability in the wing disc epithelium. We generated clones of cells mutant for *ph* and *Abd-B*, separately and simultaneously, by combining Flip-out clone induction and transgenic RNAi constructs. As presented above, wing disc cell clones which express GFP and an RNAi construct targeting *ph* only showed derepression of *Abd-B* and were expulsed as a cyst-like structure (Figure 8B–B″, arrow). Wing disc cells that express GFP and RNAi constructs targeting both *ph* and *Abd-B*, sorted-out and were extruded basally from the wing disc epithelium (Figure 8F–F″, G–G”- arrows) thus resembling the phenotype when only *ph* function is inactivated. The fact that Abd-B was not detected in the double mutant clones using immunodetection (Figure 8F′,G′, arrows) demonstrated that the *Abd-B* RNAi construct efficiently disrupted *Abd-B* expression as desired. In these images, the background signal of the Abd-B antibodies was increased so as to clearly show that there is no difference between the signal in *ph* wild-type and mutant cells. This result indicates that loss of *Abd-B* function does not impede the sorting-out of clones mutant for *ph* and, therefore, that *Abd-B* function is not necessary, or is redundant with another function, for the segregation and expulsion of the *ph* mutant cells. Therefore other targets of PH, also derepressed in *ph* mutant cells, must be implicated in the expulsion of *ph* mutant clones.

## Discussion

### Different modalities for apical and basal expulsion of *polyhomeotic* mutant epithelial cells in two *Drosophila* organs: the ovarian follicle and the wing imaginal disc

Our results establish a link between PH, a member of the PcG chromatin factors, and epithelial integrity. In two distinct tissues in *Drosophila*, the ovarian follicle and the wing imaginal disc, epithelial cell clones with lowered *ph* function (either homozygous for the *ph^504^* amorphic allele or expressing a transgenic RNAi construct targeting *ph*) are expulsed as a tight cell mass exhibiting important cell polarity and adhesion modifications. During expulsion, wild-type cells lose contacts with *ph* mutant cells and form new contacts with each other in order to maintain a normal epithelium. Significantly, when very large *ph* mutant somatic clones were recovered which covered almost an entire ovarian follicle, these clones were stably maintained in the follicular epithelium. In addition, we also observed that *ph*-overexpressing clones make smooth borders and sort-out as a cyst structure from adjacent wing imaginal disc cells expressing wild-type levels of *ph*. Therefore, the expulsion phenotype is likely due to the juxtaposition of cells with different levels of PH and consequently with different adhesive properties. Interestingly, amorphic *ph* embryos present disintegration of the ventral epidermis also suggesting a cell-cell adhesion problem [12], [13].

Expulsion of *ph* mutant cells in these two tissues is not merely a consequence of cell death since cell death markers are not expressed in these cells. In addition, the results of our clonal analysis of *ph* mutations clearly indicate that expulsion can occur independently of excessive cell proliferation. In contrast, in two recent publications, a tumor suppressor function was demonstrated for *ph* in the eye imaginal disc [16], [17] and overgrowth is also noted upon induction of *ph* mutant clones in the wing disc in one of the studies [16]. The difference with our results is likely due to the different conditions used to induce mutant clones. Indeed, the the other studies induced flipase expression was under the control of the promoter of the *eyeless* (*ey*) gene, which is expressed early and continuously during eye development. For our study, a heat shock-inducible source of flipase was used and moderate heat shock conditions gave similar *ph* mutant clone sorting out and expulsion in all the imaginal discs (Figure S1A–E). When we conducted multiple heat shocks starting early in development, we recovered rare imaginal discs with overgrowth problems (Figure S1F). Therefore our experimental conditions allow us to uncouple cell adhesion and overgrowth due to *ph* mutations and to study cell adhesion and epithelial instability independently of the severe overgrowth phenotypes. This is interesting because the loss or the perturbation of cell adhesion and polarity properties is commonly observed in advanced stages of tumorigenesis and metastasis [29], [30] and links have been established between PcG function and oncogenesis in mammals [21]. A recent report shows that mutations in *Bmi1*, a mouse PcG gene, rescue tumorigenesis involving neural stem cells by specifically controlling the adhesive capacities of these cells independently of an effect on cell proliferation [31].

Our results show that *ph* mutant clones are expulsed with very different modalities of apical versus basal expulsion. In both the wing disc and follicular epithelia, *ph* mutant cell clones can be expulsed basally and this basal expulsion involves re-organization of cell adhesion molecules and cell polarity. During progressive expulsion of *ph* mutant follicular cell clones, components of the adherens junctions, in particular DE-cadherin, delocalize from the apical to the baso-lateral domain before expulsion, in mutant cells that are still integrated within the epithelium. In the *ph* mutant cells comprising the expulsed mass, polarity and adhesion molecules are cortically relocated.

During basal expulsion in the wing imaginal disc, *ph* mutant cells reorganize in a cyst-like structure composed of a single layer of cells, which are polarized with an apical domain facing the center of the cyst and an external basal domain. The basal marker Talin accumulates at abnormally high levels as basal globs in the extruding *ph* mutant cells. Although Talin misregulation is very apparent in these clones, it does not likely provide the driving force behind expulsion since direct overexpression of Talin does not provoke any signs of cell sorting or epithelial instability.

In contrast, apical expulsion of *ph* mutant cell clones occurs in the wing imaginal disc, such that the cells find themselves lodged between the disc and peripodial membrane, and differs significantly from basal expulsion. In the case of apical expulsion, *ph* mutant cells also form a compact round cell mass, but these cells exhibit complete loss of apico-basal polarity, as evidenced by the diffuse, cytoplasmic accumulation of both apical and basal proteins. In this case, adherens junctions are disassembled between *ph* mutant and wild-type cells, but new ones do not seem to be formed between *ph* mutant cells although the cells remain in close contact. It is not possible to say at this point whether the different cell polarity defects observed are the cause or the consequence of apical vs. basal expulsion in the wing imaginal disc. Interestingly these two events, apical and basal expulsion, can occur side by side in the same region of a given disc. The cellular environment may play a role since apically-expulsed cells will find themselves closer in proximity to the peripodial membrane and will thereby be under the influence of signaling molecules like Dpp expressed by this tissue [32], [33].

It is intriguing that apical expulsion was not observed in the ovarian follicular epithelium. It is possible that adhesion between adjacent germline cells may represent a barrier to apical expulsion. Indeed, mutations in *Discs-Large* (*Dlg*), a baso-lateral marker, lead to dramatic follicle invasion by somatic cells which is aggravated if both the follicle cells and germline cells are mutant for *Dlg* [34], [35], [36]. Also, in wild-type stage 9 follicles, border cell delamination from the follicular epithelium and migration between nurse cells likely requires reduced adhesion between nurse cells [37], [38].

### PH targets and epithelial instability

PH has numerous potential target genes, as yet largely uncharacterized functionally. In polytene chromosomes of *Drosophila* salivary glands, for example, there are at least 300 different PH binding sites [39]. Several recent studies, using genome wide profiling techniques to map PcG complexes which contain PH show that in both mammals and *D. melanogaster* a large number of genes are potential PcG targets [40], [41], [42]. A high proportion of these genes encode transcriptional regulators, as well as morphogens, receptors and signaling proteins that are involved in the main developmental pathways.

Since PH is a transcriptional repressor, we were interested in identifying target genes that are derepressed in *ph* mutant cells in both the wing imaginal disc and ovary. Using immunodetection to characterize the accumulation of several adhesion and polarity molecules in *ph* mutant follicular and wing imaginal disc epithelial cells, we found that most were not affected quantitatively, but only with regards to their subcellular localization. Only the level of the basal protein Talin, was significantly increased in *ph* mutant cell clones expulsed basally from wing discs. However, since overexpression of *rhea*, the Talin-encoding gene, alone did not induce expulsion of epithelial cell clones, then excessive Talin accumulation is not likely the cause of expulsion of *ph* mutant cells in this tissue. In addition, overexpression of *ph* did not affect Talin accumulation, suggesting that *rhea* is not likely a direct target of PH.

In the wing disc, PH has been shown to repress a number of genes encoding members of known signaling pathways including *engrailed*, *hedgehog*, *decapentaplegic* [23], [43]. However, a functional link between *ph*-induced misregulation of these genes and *ph* clone expulsion in the wing disc has not been reported. In the wild-type ovary, there is no expression of *engrailed* or *hedgehog* in follicle cells [44], [45] and we found that no derepression occurs in these cells for either gene in a *ph* mutant context (data not shown). Therefore, the *engrailed* and *hedgehog* genes are PH targets in the wing disc but not in ovarian follicular cells.

Interestingly, induction of clones mutant for *thickveins*, encoding the Dpp receptor, has been shown to result in cell autonomous expulsion of the clones in both anterior and posterior wing disc compartments [46], [47], [48]. This expulsion phenotype resembles closely that associated with induction of *ph* mutant clones. However, the effect of *ph* mutations on Dpp signaling in the wing disc is non cell-autonomous upregulation of *Dpp* transcription, and this only in the anterior compartment [23], while the *ph* expulsion phenotype is cell autonomous and compartment-independent. Therefore, *ph* and Dpp signal transduction mutations likely destabilize the wing disc epithelium via different mechanisms.

In contrast, our results show that the homeotic gene *Abd-B*, a PH target in the embryo and wing imaginal disc, is also a target of PH in ovarian follicular cells [22]. We found that *Abd-B* is not expressed in any of the different somatic cell types or in the germline in the adult *Drosophila* ovary. Interestingly, in the embryo, among the mesodermal somatic cells that coalesce with germ cells to form the primitive gonad, the anterior-most do not express *Abd-B*, while the posterior-most do [49]. However, among these precursors, which will give rise to the follicular cells in the adult ovary is not known. We show that lowering *ph* function in ovarian follicular cells (by inducing clones either with the amorphic *ph^504^* allele or an RNAi transgene targeting *ph*) leads to ectopic expression of *Abd-B* in these cells indicating that *ph* wild-type function represses *Abd-B* expression in these adult ovarian somatic cells.

Since *Abd-B* is common target of PH in both the ovary and wing disc, we induced *Abd-B* overexpression clones and found that these are round with smooth borders, though not efficiently expulsed from the epithelial layer. *Abd-B* overexpression therefore does not produce a perfect phenocopy of *ph* loss of function. Indeed, results of our epistatic analysis also indicate that *Abd-B* derepression is not necessary for *ph*-induced expulsion. Therefore, expulsion of *ph* mutant cells does not depend solely on the derepression of *Abd-B*. Other target genes or pathways deregulated by *ph* mutations may be involved in *ph*-associated epithelial instability, like *Ultrabithorax* [22] or the Notch or JAK-STAT pathways, which have been implicated in overproliferation induced by *ph* mutations in the eye imaginal disc [16], [17].

It is nonetheless noteworthy that *Abd-B* overexpression clones sort out from an epithelium that does not express *Abd-B*. Indeed, formation of straight segmental boundaries during development in *Drosophila* and vertebrates has been attributed to differential Hox gene expression, though establishing the specific link to cell adhesion molecules that are differentially expressed across these boundaries has proven difficult [50], [51]. One recent study was able to determine a link between *Abd-B* and cell adhesion and polarity proteins governing morphogenesis of the posterior external respiratory organ of the larva, the spiracles. The authors show that Abd-B activates JAK-STAT signaling and three transcription factors, *spalt*, *cut*, *empty spiracles* which in turn activate regulatory molecules of actin cytoskeleton, cell adhesion and cell polarity [52]. It would be interesting to determine whether *Abd-B* misregulation in the ovarian follicle or wing imaginal disc also impacts on the four *Abd-B* targets in spiracle formation.

### Other PcG proteins

Since *ph* has been shown to regulate target gene expression during development via its interactions with other *PcG* genes, then *PcG* genes besides *ph* may also be implicated in epithelial integrity. In the wing imaginal disc epithelium, homozygous mutant clones for both *Psc* and the related adjacent *Su(z)2* gene lead to an expulsion phenotype resembling that of *ph* mutant clones, while this was not the case for other *PcG* genes tested, notable *Asx*, *E(z)*, *Pc*, *Pcl*, *Sce* and *Scm* [22]. Correlated with this, *ph* and *Psc-Su(z)2* mutant clones showed rapid derepression of *Abd-B* and *Ubx* upon clone induction, while the others showed either no derepression of these two homeotic genes (the case for *Asx* and *E(z)*) or an important lag time before homeotic gene deregulation (the case for *Pc*, *Pcl*, *Sce* and *Scm*). These results have been interpreted as indicating specific function for *ph* and *Psc/Su(z)2* in transcriptional repression of homeotic target genes (hence the rapid effects on expression of these targets), while the other *PcG* gene functions would be required for long-term epigenetic marking of target genes for maintenance of repression through cell divisions. In addition, for a number of different *PcG* genes, notably *Pc* and *Pcl*, studies have shown that induction of mutant clones results in the recovery of vesicles within leg and wing tissue in the adult indicative of expulsion of cell clones [53], [54], [55]. These results suggest that, given enough time to affect epigenetic marks on target genes by removing certain *PcG* gene function, the expulsion phenotype may be recovered for a large number of *PcG* genes.

We conducted a similar clonal analysis of *PcG* gene mutant clones in the ovarian follicular epithelium, and, like in the wing disc, *ph*, but not *Asx*, *Pc*, *Pcl*, *Sce* or *Scm* presented an expulsion phenotype. *Psc-Su(z)2* mutant cell clones, on the other hand, which are expulsed from the wing disc like *ph* mutant clones [22], did not present the expulsion phenotype in the follicular epithelium. These results may be explained by the important differences between the development and fate of wing disc and follicular cells. The wing disc epithelium is established during embryogenesis, undergoes many rounds of cell division during larval development and is present until eclosion, while follicular cells have a much more limited division potential and a shorter lifetime corresponding to the development of an individual egg chamber during oogenesis. Indeed, comparing the different results obtained between the wing disc and follicular epithelia when the clonal *ph* RNAi approach was used is informative. While in the wing disc, reduction of *ph* levels by RNAi is always accompanied by *Abd-B* derepression, *Abd-B* derepression was not observed in the totality of the follicular cell clones indicating less efficient kinetics of derepression of *Abd-B* in this tissue. In addition, since follicular cells die by apoptosis at the end of oogenesis, it is not possible to assay the effects of long term absence of PcG proteins as has been done in the wing tissue. Taken together, our results and those from other laboratories suggest a distinct direct action of PH on target gene expression compared to that of other PcG members, though these proteins may be present together in complexes on some of the same chromosomal sites.

## Materials and Methods

### Fly stocks

Flies were raised at 25°C on standard medium. The *ph^504^* (noted *ph^0^*) amorphic allele inactivates both the *ph-p* and *ph-d* units [13]. The other *Polycomb* Group mutant strains analyzed, *Scm*, *Sce*, *Pcl*, *Asx*, *E(z)*, *Psc* and *Su(z)2* have been described [22]. The RNAi strains *UAS-ph IR* (50027 line) and *UAS-Abd-B IR/TM3 Sb* (12024 line) were obtained from the Vienna Drosophila RNAi Center (VDRC) [56]. The reporter constructs *shotgun-lacZ* (*shg^P34-1^*) and *armadillo-lacZ*, and the overexpression strain *yw hsp-flp;;UAS-rhea* were a gift from J.R. Huynh [57], [58]. M. Van Doren kindly provided the overexpression line *UAS-Abd-B*. The *UAS-cDNA-ph* (*m20* and *m35*) strains were provided by S. Netter [59].

### Clonal analysis

In the ovaries, mutant clones for *ph* were generated by mitotic recombination using the FLP-FRT system [27] in females *hs-flp tub-lacZ FRT101/ph^504^ w FRT101* or *ubi-nls-GFP FRT101/ph^504^ w FRT101*; *hsflp^38^/+*. *hs-flp tub-lacZ FRT101* and *ubi-nls-GFP FRT101*; *hs-flp^38^* lines were gifts from S. Goode (unpublished) and A. Guichet, respectively; the *ph^504^ w FRT101* was previously described [22]. Induction of Flipase expression was done by heat-shocking females just after eclosion at 38°C for 1 hour. Flies were dissected 2, 4 or 8 days after eclosion. Clones were detected by the loss of *lacZ* or *GFP* expression. In imaginal discs, mitotic recombination clones homozygous for *ph^504^* were also induced by the FLP-FRT technique, by incubation of larvae at 37°C for 1 hour, 48 hours after egg laying (AEL). L3 stage larvae were dissected 5 days AEL. Different conditions for heat shock-induced flipase expression were also used in some experiments (Figure S1) which involved using a *hs-flp* transgenic construct present on the X chromosome (P{hsFLP}122), conducting heat shocks earlier at 24 hours AEL and multiplying heat shocks, in particular, three heat shocks, the first between 24 and 38 hours, the second between 48 and 62 hours and the third between 72 and 86 hours AEL.

Somatic overexpression was achieved by generating Flip-out/Gal4 clones [60]. *hsp-flp^70^;Tub-FRT CD2 FRT-Gal4 UAS-nls-GFP/TM3 Sb* (a gift from J. Silber) flies were crossed with transgene line of interest *UAS-x*. Clones expressing Gal4 were induced by ‘flipping out’ an interruption cassette Tub>CD2>Gal4 transgene in a genetic background that contained the *UAS-x* constructs, as well as a *UAS-GFP* transgene. Thus, the co-expressing GFP marks cells that express the *UAS-x* constructs. For the wing imaginal discs, larvae were heat-shocked for 1 hour at 37°C, 48 hours after egg laying and dissected at L3 stage 5 days after egg laying. For the ovaries, females were heat-shocked 1 hour at 38°C at eclosion and dissected 6 days after eclosion.

### Antibody staining

Adult ovaries were fixed and stained as described [25]. Immunocytochemistry of wing imaginal discs was performed as described [27]. Primary antibodies were: rat anti-DE-cadherin DCAD2 (1/50, Developmental Studies Hybridoma Bank), mouse anti-Armadillo N2 7A1 (1/50, DSHB), mouse anti-Integrin βPS CF.6G11 (1/50, DSHB), mouse anti-Talin C19 (1/50, J.R. Huynh), mouse anti-Abdominal-B 1A2E9 (1/50, DSHB), rabbit anti-Bazooka (1/500), rabbit anti-PKC (1/500), mouse anti-Hts RC (1/100, DSHB), mouse anti-β-Galactosidase 40-1A (1/50, DSHB), mouse anti-GFP (1/200, Roche), rabbit anti-GFP (1/500, FluoProbes). F-actin was labelled with rhodamine-phalloidin (1/100, FluoProbes). DNA was stained with DAPI (Sigma), Propidim iodide (Invitrogen) or TO-PRO-3 (Invitrogen). Fluorescence-conjugated secondary antibodies were purchased from Jackson Immunoresearch and Invitrogen and were used at a 1/200 dilution. All samples were mounted in citifluor (Biovalley). Samples were examined with a Leica TCS SP2 confocal microscope and composite figures were prepared using Adobe Photoshop CS2, Metamorph and ImageJ.

## Supporting Information

Figure S1 *ph* loss of function clones sort-out and are extruded from both wing and eye-antenna imaginal discs and overgrowth occurs using stronger conditions for clonal induction. (A) Mosaic eye-antenna imaginal disc in which *ph^504^* homozygous mutant clones of cells, which are marked by ectopic expression of *Abd-B* have been induced via mild heat shock induction of a hspprom-flipase transgene (one heat shock at 48 h AEL). (B,C) Mosaic eye-antenna imaginal discs in which clones of cells have been induced via similar mild heat shock induction of a hspprom-flipase transgene expressing *GFP* ectopically (B′,B″- see arrow for example) or *GFP* and a RNAi construct targeting *ph* (C′,C′- arrow). In these images, the antennal disc is to the left and the eye disc is to right oriented with posterior to the right. The clones expressing only *GFP* ectopically form wiggly borders with adjacent non *GFP*-expressing cells (B′,B″-arrow) and are integrated normally in the wing disc epithelium as evidenced by the regular spacing of nuclei stained with DAPI (B - arrow), whereas clones expressing both *GFP* and the RNAi construct against *ph* are of approximately the same size, but are round and form smooth borders with neighboring wild-type (C′,C″-arrow) and are segregated from wild type cells as evidenced by the grouping together of nuclei stained with DAPI (C-arrow). (D) Mosaic wing imaginal disc in which *ph^504^* homozygous mutant clones of cells, which are marked by ectopic expression of *Abd-B* (D′,D″-arrows), have been induced via earlier heat shock induction of a hspprom-flipase transgene (24 hAEL). DAPI marks all the nuclei. The wing disc is oriented with ventral towards the top and anterior to the left. (E,F) Mosaic eye-antenna imaginal discs in which *ph^504^* homozygous mutant clones of cells, which are marked by ectopic expression of *Abd-B* (E,E′-arrows), or not marked (F-rightmost arrow) have been induced via repeated heat shock induction of a hspprom-flipase transgene (three heat shocks 24 h apart starting at 24 h AEL). The eye-antenna discs are oriented with the antenna disc to the left and the eye disc to the right oriented with posterior towards the right. In F, the leftmost arrow indicates the antenna disc and the middle arrow indicates the wing disc. In eye-antenna imaginal discs (as well as all wing and leg imaginal discs, data not shown), these stronger heat shock conditions allow recovery of rare *ph* mutant clones with a clear overgrowth phenotype (E,E′-arrows and F-rightmost arrow).
(1.63 MB TIF)
