# Supplementary figures and images for: Mutations in the Polycomb Group Gene polyhomeotic Lead to Epithelial Instability in both the Ovary and Wing Imaginal Disc in Drosophila
Source: PLoS One. 2010 Nov 11;5(11):e13946. doi: 10.1371/journal.pone.0013946 (PMC2978711; doi:10.1371/journal.pone.0013946)

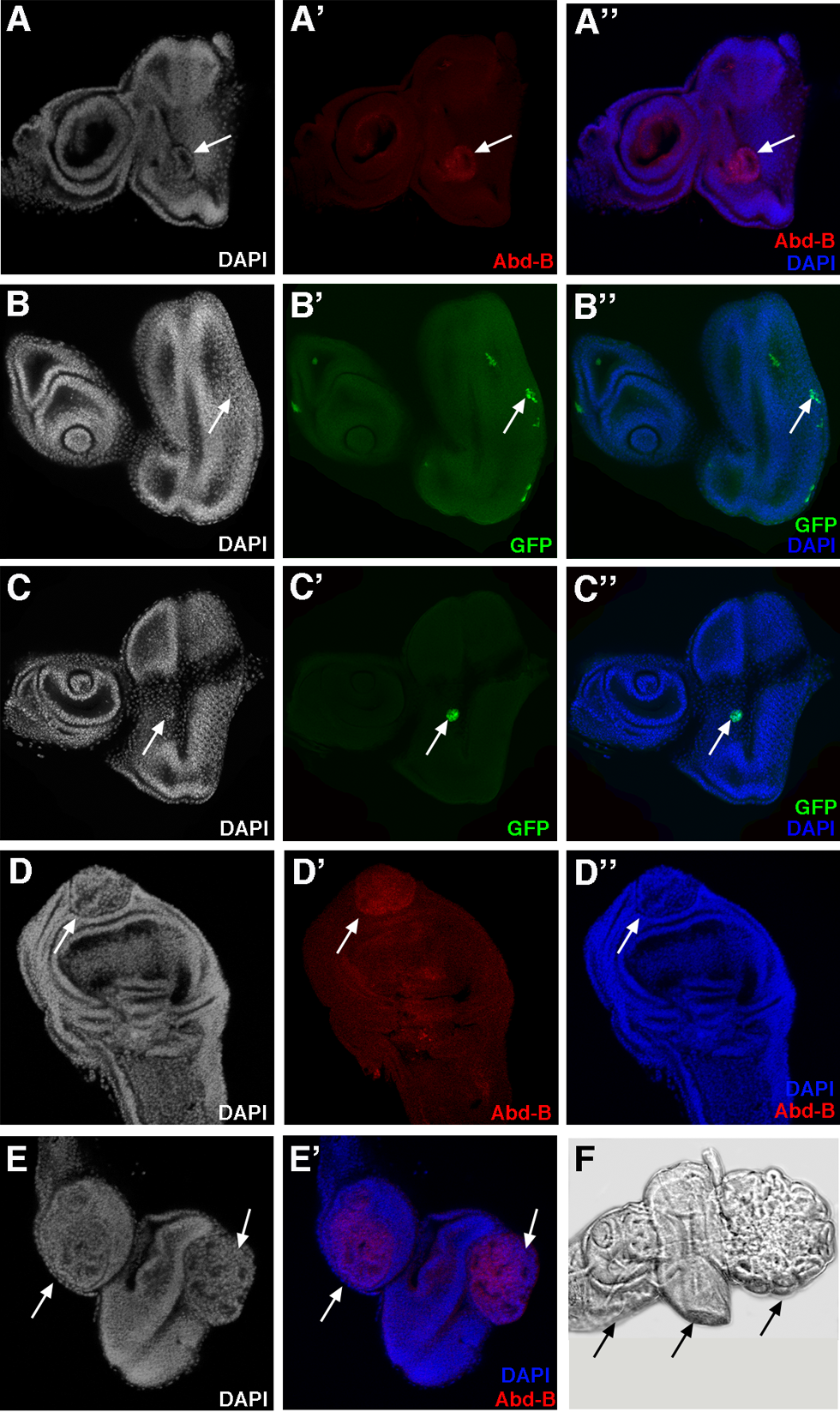

Supplement: Figure S1 — ph loss of function clones sort-out and are extruded from both wing and eye-antenna imaginal discs and overgrowth occurs using stronger conditions for clonal induction. (A) Mosaic eye-antenna imaginal disc in which ph504 homozygous mutant clones of cells, which are marked by ectopic expression of Abd-B have been induced via mild heat shock induction of a hspprom-flipase transgene (one heat shock at 48 h AEL). (B,C) Mosaic eye-antenna imaginal discs in which clones of cells have been induced via similar mild heat shock induction of a hspprom-flipase transgene expressing GFP ectopically (B′,B″- see arrow for example) or GFP and a RNAi construct targeting ph (C′,C′- arrow). In these images, the antennal disc is to the left and the eye disc is to right oriented with posterior to the right. The clones expressing only GFP ectopically form wiggly borders with adjacent non GFP-expressing cells (B′,B″-arrow) and are integrated normally in the wing disc epithelium as evidenced by the regular spacing of nuclei stained with DAPI (B - arrow), whereas clones expressing both GFP and the RNAi construct against ph are of approximately the same size, but are round and form smooth borders with neighboring wild-type (C′,C″-arrow) and are segregated from wild type cells as evidenced by the grouping together of nuclei stained with DAPI (C-arrow). (D) Mosaic wing imaginal disc in which ph504 homozygous mutant clones of cells, which are marked by ectopic expression of Abd-B (D′,D″-arrows), have been induced via earlier heat shock induction of a hspprom-flipase transgene (24 hAEL). DAPI marks all the nuclei. The wing disc is oriented with ventral towards the top and anterior to the left. (E,F) Mosaic eye-antenna imaginal discs in which ph504 homozygous mutant clones of cells, which are marked by ectopic expression of Abd-B (E,E′-arrows), or not marked (F-rightmost arrow) have been induced via repeated heat shock induction of a hspprom-flipase transgene (three heat shocks 24 h apart starting [file pone.0013946.s001.tif]
